# Reverse Genetics for Functional Genomics of Phytopathogenic Fungi and Oomycetes

**DOI:** 10.1155/2009/380719

**Published:** 2009-10-07

**Authors:** Vijai Bhadauria, Sabine Banniza, Yangdou Wei, You-Liang Peng

**Affiliations:** ^1^The MOA Key Laboratory of Molecular Plant Pathology, Department of Plant Pathology, China Agricultural University, Beijing 100193, China; ^2^Department of Biology, University of Saskatchewan, Saskatoon, SK, Canada S7N 5E2; ^3^Crop Development Centre, University of Saskatchewan, 51 Campus Drive, Saskatoon, SK, Canada S7N 5A8

## Abstract

Sequencing of over 40 fungal and oomycete genomes has been completed. The next major challenge in modern fungal/oomycete biology is now to translate this plethora of genome sequence information into biological functions. Reverse genetics has emerged as a seminal tool for functional genomics investigations. Techniques utilized for reverse genetics like targeted gene disruption/replacement, gene silencing, insertional mutagenesis, and targeting induced local lesions in genomes will contribute greatly to the understanding of gene function of fungal and oomycete pathogens. This paper provides an overview on high-throughput reverse genetics approaches to decode fungal/oomycete genomes.

## 1. Introduction

**Figure 1:**
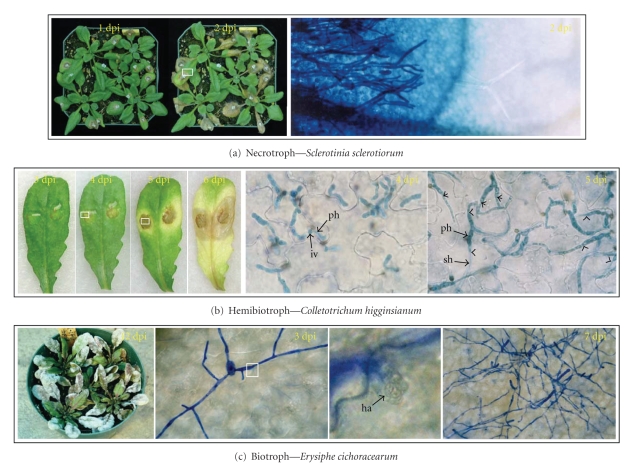
*Arabidopsis*—fungus interaction. (a) Necrotrophic interaction—*Sclerotinia sclerotiorum* infects *Arabidopsis* by killing host tissues in advance of hyphal colonization. Trypan blue stains necrotic dead tissues. (b) Hemibiotrophic interaction—*Colletotrichum higginsianum* initiates infection process by a biotrophic phase at 3–4 days post-inoculation (dpi), which is associated with infection vesicles (iv) and large primary hyphae (ph). Thin secondary hyphae (sh) appear in the necrotrophic phase at 5 dpi. (c) Biotrophic interaction—*Erysiphe cichoracearum* colonizes the plant surface by producing haustorium (ha) into epidermal cells for nutrient uptake and keeps the host cell in survival till the pathogen completes its life cycle by conidiation.

Fungal and oomycete phytopathogens are major constraints in global food production as they cause many of the world's most notorious plant diseases. These phytopathogens can be broadly divided into those that kill the host and feed on the cell contents (necrotrophs), those that require a living host to complete their life cycle (biotrophs), and those that act as both biotrophs and necrotrophs at different stages of infection (hemibiotrophs). Figure 1 illustrates three phytopathosystems (necrotrophic *Arabidopsis*-*Sclerotinia sclerotiorum*, biotrophic *Arabidopsis*-*Erysiphe cichoracearum,* and hemibiotrophic *Arabidopsis*-*Colletotrichum higginsianum*), which have been widely researched with the objective to understand molecular mechanisms underpinning pathogen development and pathogenesis.

With the advent of high-throughput sequencing technologies, the number of fungal genomes sequenced is increasing rapidly. More than 40 fungal genomes have now been sequenced, of which 12 are plant pathogens: *Botrytis cinerea* (necrotroph, grey mould of grape, and other host species), *Sclerotinia sclerotiorum* (necrotroph, white mould of many host species), *Fusarium graminearum *(hemibiotroph, cereal head blight), *Fusarium oxysporum *(hemibiotroph, wilt diseases of many host species), *Fusarium verticillioides *(hemibiotroph, seed rot of corn), *Magnaporthe oryzae *(hemibiotroph, rice blast), *Nectria haematococca *(hemibiotroph, pea wilt), *Mycosphaerella fijiensis *(hemibiotroph, black leaf streak of banana), *Mycosphaerella graminicola *(hemibiotroph, leaf blotch of wheat), *Puccinia graminis *(biotroph, cereal rust), *Stagonospora nodorum *(necrotroph, wheat glume blotch), and *Ustilago maydis *(biotroph, corn smut). In addition, the genomes sequences of two oomycete pathogens have also been sequenced: *Phytophthora ramorum *(hemibiotroph, sudden oak death) and *Phytophthora sojae *(hemibiotroph, stem/root rot of soybean) [1].

In contrast to “structural genomics,” where the entire nucleotide sequence of an organism's genome is determined, “functional genomics” encompasses genome-wide experimental approaches to assess gene function by making use of the information and reagents provided by “structural genomics” [2]. With the exponential progress in structural genomics, the major undertaking in fungal/oomycete genomics is now to investigate the function of the large number of genes identified by* in silico *approaches. Reverse genetics, an approach to discover the function of genes, will contribute greatly in deciphering the molecular mechanisms underlying fungal/oomycete development (sporulation, spore germination, appressorium or infection structure formation, appressorium morphogenesis and penetration), fungal nutrition (uptake of nutrients, e.g., iron, phosphorous from the host milieu) and the interactions between fungi/oomycetes and their host plants (compatible or incompatible interactions). For the longest time, oomycetes were considered to belong to the fungi based on certain morphological similarities. Both fungi and oomycetes are characterized by filamentous vegetative growth, the production of mycelia, and formation of spores through asexual and sexual processes. Similarities also exist in infection structures and the mode of infection. However, cell walls of oomycetes primarily consist of cellulose rather than chitin, which is the main component of true fungal cell walls. Furthermore, hyphae of oomycetes are nonseptate and are diploid in their vegetative form, which distinguishes them from true fungi. Based on morphological differences and molecular evidence, it was found that oomycetes were related to heterokont algae and thus they were reclassified under the stramenopiles [3, 4]. Among the oomycetes, *Phytophthora*, *Pythium*, and *Peronospora* are well-known pathogens of high economic significance. These oomycete phytopathogens are equally destructive as fungal phytopathogens. Unless specified otherwise, fungal and oomycete phytopathogens will be referred to as phytopathogens hereafter in the paper.

Reverse genetics approaches have been extensively employed to unveil infection strategies of necrotrophic and hemibiotrophic phytopathogens at the molecular level. Reverse genetics cannot be applied to obligate biotrophs because suitable methods for the molecular analyses, such as in vitro cultivation, transformation, and gene disruption methods, are lacking. This has hindered the identification of corresponding genes in these species.

This paper provides an overview of reverse genetics approaches, such as targeted gene disruption/replacement (knock-out), gene silencing (knock-down), insertional mutagenesis, and targeting induced local lesions in genomes (TILLING), which can be applied to phytopathogens in order to determine gene function.

## 2. Reverse Genetics Approaches

### 2.1. Targeted Gene Disruption/Replacement (Knock-Out)

One of the most powerful approaches for dissecting gene function in phytopathogens is the study of the phenotypes of mutants in which a genomic locus has been altered by insertion (gene disruption) or replacement (gene replacement) with heterologous DNA [5–7]. Such reverse genetic approaches have become even more straightforward since increasing amounts of genomic sequence information have become available for a rising number of phytopathogen species.

**Figure 2:**
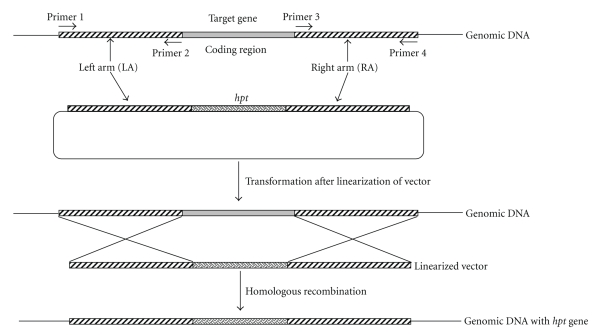
Schematic representation of a gene replacement strategy. The 5′- and 3′- flanking regions (left and right arms) of the target gene are PCR amplified from genomic DNA of wild-type strain and ligated into vector containing the hygromycin phosphotransferase (*hpt*) gene. This vector is then linearized using restriction enzyme and introduced into the protoplast of the wild-type strain through transformation. Homologous recombination replaces the gene of interest with *hpt* gene.

The targeted integration of DNA constructs by homologous recombination enables inactivation of genes by disruption, deletion, or replacement. Homologous recombination involves a reciprocal exchange of DNA sequences as found between two chromosomes that carry the same genetic loci. Homologous recombination between a target gene and the introduced DNA carrying its mutant allele results in targeted gene knock-out. This approach was first pioneered for the budding yeast *Saccharomyces cerevisiae *to decipher gene function [8]. Since then, it has been applied to several phytopathogens. Four types of gene knock-out approaches are used most commonly to elucidate the function of genes: simple disruption of the gene of interest by a resistance cassette; alteration of the expression of the candidate gene rather than its deletion; over-expression or miss-expression of a gene; gene replacement, which combines the second and third approaches. A targeted gene replacement strategy is shown in Figure 2.

Kämper [9] developed a PCR-based technique to generate gene replacement mutants in *Ustilago maydis*. In this technique, the 5′ and 3′-regions of the target gene are first amplified by PCR, and then ligated directionally to a marker cassette via two distinct *Sfi*I restriction sites, providing the flanking homologies needed for homologous recombination. The ligation product is then used as a template to amplify the replacement construct, which can be used directly for transformation of *U. maydis*. Brachmann et al. [10] described a versatile reverse genetics approach based on the technique developed by Kämper [9] to generate mutants of *U. maydis*. They constructed a comprehensive collection of insertion cassettes with the basic structure of an optional reporter gene (green fluorescent protein [*gfp*] gene), a resistance cassette (*hpt *gene), and an optional promoter cassette flanked by two *Sfi*I recognition sites for PCR amplified gene fragment insertion. Using this approach, the authors generated two replacement mutants [10]. In both mutants, the endogenous promoter of the pheromone gene *mfa1 *drives expression of the *gfp *gene. Simultaneously, expression of the *mfa1 *open reading frame (ORF) is modulated either by the carbon source-regulated *crg1* promoter or the nitrogen source-regulated *nar1 *promoter. The expression was induced by pheromone, and pheromone expression was only observed when the heterologous promoters were active. Cho et al. [11] developed a method for high-throughput targeted gene disruption for *Alternaria brassicicola *using linear minimal element (LME) constructs. *Alternaria brassicicola *causes black spot disease of cultivated Brassicaceae species and has been used frequently as a necrotrophic fungal pathogen for studies with *Arabidopsis*. To improve targeted gene disruption efficiency as well as to expedite gene disruption construct production, the authors used a short linear construct with minimal elements, an antibiotic resistance selectable marker gene, and a partial target gene. Using LME constructs, targeted gene knock-out efficiency was improved substantially compared with standard plasmid construct.

Targeted gene disruption has been applied to many phytopathogens, including *M. oryzae* [12–23], *Colletotrichum gloeosporioides *(causal agent of anthracnose disease) [24, 25], *U. maydis* [9, 10], *A. brassicicola *[11], *Cochliobolus carbonum *(northern corn leaf spot) [26],* and F. graminearum* [27]. Over the past decade *M. oryzae *(formerly *Magnaporthe grisea*) has emerged as a seminal model to elucidate mechanisms underlying fungal development, fungal-plant interactions, and pathogenicity and virulence. Using targeted gene disruption, many genes implicated in virulence and pathogenicity of the rice blast fungus have been characterized. Some of these pathogenicity genes are *MPG1* (class I hydrophobin) [12], *PMK1* (homologue of yeast *Fus3/Kss1* MAP kinase) [13], *MAC1 *(adenylate cyclase) [14], *Mps1 *(homologue of yeast *Slt7* MAP kinase kinase) [15], *ABC1 *(ATP-binding cassette 1) [16], *GAS1* and *GAS1* (homologues of *gEgh16 *of the powdery mildew fungus) [17], *ACE1* (avirulence conferring enzyme 1) [18], *CHM1* and *MST20 *(p21-activated kinases) [19], *MMT1 *(metallothionein 1) [20], *ATG1* (serine and threonine kinase) [21], *CUT2 *(cutinase 2) [22], *DSE1* (defense suppressor 1) [23], and *COM1 *(conidial morphology 1) [Dr. You-Liang Peng, The MOA Key Laboratory of Molecular Plant Pathology, China Agricultural University, Beijing, China (personal communication)].

Inserting a selectable marker gene like *hpt* into the gene of interest requires several restrictions, ligation, cloning and subcloning steps, and is time consuming. It makes this classical DNA recombinant technology inefficient for large-scale functional analyses of genes. To circumvent these limitations, Invitrogen has introduced the Gateway in vitro recombination cloning technology. Using this technology, high-throughput cloning of target genes for knock-out and knock-down (discussed in the next section) can now be achieved. The Gateway cloning system exploits the precise, site- specific recombination system utilized by bacteriophage lambda in order to shuttle DNA fragments between entry and destination vectors bearing compatible recombination sites while maintaining the ORF [28]. This technology allows researchers to construct multiple destination vectors for different purposes, such as expression in *Escherichia coli* or *S. cerevisiae*, from a single entry vector with inserted gene of interest [29]. Since its inception in functional genomics analyses, GATEWAY technology has been widely used to characterize many genes. Recently, Shafran et al. [28] developed two high throughput functional genomics tools for filamentous fungi based on the GATEWAY technology, namely, pTroya (Knock-down) and Gene Blast (Knock-out). The authors tested the utility of both tools on *Colletotrichum gloeosporioides* for loss of function analyses.

A major advantage of gene knock-out is its propensity to target a specific genetic region. However, the value of this conventional approach for generating knock-out strains is limited by its poor efficiency for homologous recombination (which varies considerably among phytopathogen species) due to nonhomologous (ectopic) integration of the transforming DNA, and the time required for construction of replacement vectors. Higher recombination efficiencies can be obtained by increasing the length of homologous DNA flanking the transformation marker, albeit this is a tedious process when standard molecular techniques are used for the construction of gene replacement cassettes. The majority of fungi consist of multicellular and/or multinuclear hyphae, and some of them have two or more genetically different nuclei in a common cytoplasm (heterokaryon). These characteristics of fungi make gene targeting complicated and inefficient.

### 2.2. RNA Interference (Knock-Down)

**Figure 3:**
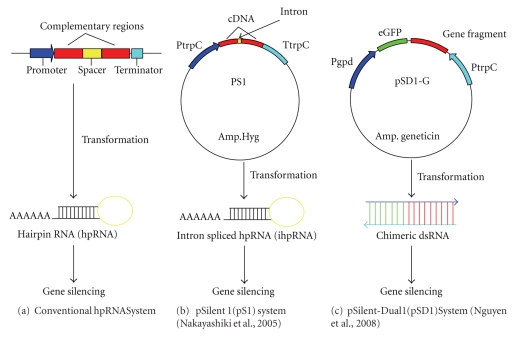
RNAi strategies: (a) conventional hpRNAi, transcripts with complementary or near-complementary 20- to 50-base pair inverted repeats form double-stranded RNA (dsRNA) hairpins. These dsRNAs are processed into micro-RNAs that mediate mRNA degradation. (b) pSilent1 (heterogeneous nuclear RNA expressing vector system), it carries a hygromycin resistance cassette and a transcriptional unit for hairpin RNA expression with multiple cloning sites and a spacer of an intron sequence. (c) pSilent-Dual1 system (opposing dual promoter system), trpC and gpdA promoters were cloned in a convergent manner separated by multicloning site. For co-silencing, a 0.41 kb eGFP fragment was first inserted in pSD1, resulting in pSD1-G. Gene fragments of ~500 bp can be inserted into pSD1 vector, which will express corresponding chimeric double-stranded RNA, a template for homology-dependent degradation of the target mRNA.

RNA interference-(RNAi-) based gene silencing (post-transcriptional gene silencing in plants and RNAi in animals) is an exciting strategy for reverse genetics [30]. RNAi is a technique in which double-stranded RNA (dsRNA) triggers the degradation of a homologous mRNA, thereby diminishing or abolishing gene expression. It was first discovered in the nematode *Caenorhabditis elegans *as a response to dsRNA, which resulted in sequence-specific gene silencing [31]. This technique is based on the generation of dsRNA molecules, which act as templates for Dicer ribonuclease III producing short interfering RNAs (siRNAs). These siRNAs are incorporated into the RNA-inducing silencing complex (RISC) and serve as sequence-specific guides that target corresponding mRNA molecules for destruction [32, 33]. RNA-mediated gene silencing methods that block the expression of genes at the post-transcriptional level have been identified in a few economically important phytopathogens like *M. oryzae* [34] and *P. infestans* [35–37]. The most common forms involve the introduction of antisense RNA, dsRNA, or sense transgenes (also called co-suppression in plants or quelling in fungi). RNAi is an important tool not only for elucidating the function of the many unknown genes but also for the identification of genes essential for phytopathogenic growth and pathogenesis [38]. The application of RNAi in phytopathogens is limited to a few species. Figure 3 illustrates three RNAi strategies (conventional hairpin RNAi, intron spliced hairpin RNAi, and chimeric double stranded RNA mediated silencing) that have been used in functional analyses of phytopathogens to date.

Kadotani and colleagues [34] carried out a systematic analysis of RNA silencing in the blast fungus *M. oryzae *using expression of the enhanced green fluorescence protein (eGFP) gene as a reporter. They found that the accumulation of eGFP mRNA was drastically reduced in the silenced transformants. In addition, it was noticed that small interfering RNAs (siRNAs) were present only in the silenced transformants. These results indicated that RNA silencing operated in *M. oryzae*, which provided a new tool for genome-wide functional analysis of this fungus. Akihiro and colleagues [39] demonstrated the applicability of this targeted gene silencing as a useful reverse-genetics approach in *Bipolaris oryzae*, causal agent of brown leaf spot disease in rice. A polyketide synthase gene (*PKS1*) implicated in fungal melanin biosynthesis was targeted by gene silencing as a marker. The silencing vector encoding hairpin RNA (hpRNA) of the *PKS1 *fragment was constructed and introduced into the *B. oryzae *genomic DNA (Figure 3(a)). Silencing of the *PKS1 *gene resulted in reduction of *PKS1 *mRNA expression and albino phenotypes. More recently, Nguyen and colleagues [40] described a novel high-throughput approach for gene function analysis using RNAi, which provides an alternative to the gene knock-out by homologous recombination. The authors developed an RNA silencing vector, pSilent-Dual1 (pSD1) that carries two convergent dual promoters, the *Aspergillus nidulans* tryptophan promoter (*PtrpC*) and the *A*. *nidulans* glyceraldehyde-3-phosphate dehydrogenase promoter (*Pgpd*) (Figure 3(c)). Both promoters have been used to drive constitutive gene expression in a large number of filamentous fungi. A multicloning site (MCS) has been inserted between two promoters. The greatest merit of the pSD1 system over others, such as hpRNA or intron spliced hair-pin RNA (ihpRNA) (Figure 3(b)) silencing system is that it allows a single step cloning for generation of an RNAi construct. To facilitate efficient screening for silenced transformants, Nguyen and colleagues [40] incorporated the *gfp* gene into pSD1 system. It allows expression of a chimeric RNA and assessment of gene silencing efficiency by utilizing a recipient strain that produces GFP and therefore fluoresces green when using epifluorescence microscopy. A main bottleneck of this system is its lower silencing efficiency compared with hpRNA or ihpRNA-expressing RNA-silencing vectors. Formation of dsRNA in the pSD1 system requires physical annealing of two different RNA molecules in the target cells while that in the hpRNA systems is achieved by self-folding of inverted repeats within RNA molecule. The difference in dsRNA formation between the systems can be a major cause of the different silencing efficiencies. The authors generated a series of knock-down mutants of almost all known calcium related genes in the genome of *M. oryzae* and examined for phenotypical defects.

Gene knock-down requires relatively short stretches of sequence information. This is a major advantage for phytopathogens for which there is little sequence information available. As RNAi works at the mRNA level, its efficacy is not compromised by the presence of nontransformed nuclei or multicopy genes due to aneuploidy [41]. RNAi causes only a partial reduction in, but not a complete loss of, gene expression. Partial gene suppression is considered a main drawback of RNAi. However, it could be a merit where the effect of an essential gene on a phenotype is of interest. Gene knock-down offers a more convenient and effective tool, especially in combination with an inducible promoter that allows gene expression to be diminished at specific stages during development [42]. Another disadvantage of gene knock-down is that, as it requires only a short sequence, genes other than those targeted might be silenced. This causes unexpected changes in gene expression patterns (off-target effects). Testing for the possibility of off-target effects is simpler for phytopathogen species for which complete genome sequence data are available but remains elusive for those phytopathogens whose genomes have not been sequenced [41].

### 2.3. Insertional Mutagenesis

Insertional mutagenesis is a powerful tool to dissect the molecular mechanism of most genetically determined processes, including those in phytopathogens. Its main advantage is that it does not require a priori genome information. Therefore, it can be applied to those phytopathogens, whose genomes have not been sequenced yet. Classical genetic analysis approaches using mutagens such as chemicals or ultraviolet light have yielded a wealth of information on pathogen development and pathogenesis. These mutagens normally generate base pair deletions or substitutions, which can result in the loss or an alteration of gene function, and their relative lack of specificity allows saturation of a genome with mutations. Subsequent genetic analysis of mutant strains can, however, be time consuming because it usually encompasses isolation of the mutated gene by complementation using a genomic DNA library from the wild-type strain. This partly explains the increasing use of insertional mutagenesis by chromosomal integration of transforming DNA. The presence of a selectable marker in the transforming DNA can be used to establish linkage between the insertion and the observed phenotype, and to recover DNA representing the mutated allele for cloning and subsequent analyses [43].

Biocomputational analyses of sequenced genomes have extracted only a handful of genes. At this point, the challenge is to convert the available plethora of genomic sequences into meaningful biological information, which will require the large-scale construction of mutant libraries. Therefore, insertional mutagenesis techniques like *Agrobacterium tumefaciens*-mediated transformation (ATMT) and restriction enzyme mediated integration (REMI) have been developed.

Insertional mutagenesis by ATMT has been well established in the recent years. In the filamentous fungi, transformation with DNA that does not exhibit homology with the fungal genome results in heterologous integration of transforming DNA into the genome, which makes it possible to use the transforming DNA as an insertional mutagen to disrupt genes, and eventually assist in the study of plant disease [44]. *Agrobacterium tumefaciens* is a plant pathogenic bacterium capable of causing crown gall tumors on plants by transferring a part of its DNA (Transfer DNA; T-DNA), located on the tumor inducing plasmid, through a type IV secretion system to the hosts. Once inside the host, the T-DNA is targeted to the nucleus where it randomly integrates into the host genome. A full description of ATMT is beyond the scope of this paper, and for detailed description on ATMT, the reader is referred to Michielse et al. [45, 46]. ATMT has been widely exploited for transforming a number of phytopathogen species, such as *Botrytis cinerea* [47], *Colletotrichum* spp. (*C. gloeosporiodes*,* C. lagenarium* and* C. trifolii*) [48–50], *Fusarium* spp. (*F. circinatum* [51] and *F. oxysporum* [52]),* Leptosphaeria *spp. (*L. maculans* and* L. biglobosa*) [53],* M. oryzae* [52, 54, 55], *Mycosphaerella graminicola* [56], *Venturia inaequalis* [57], *Pythium ultimum* [58], and *Phytophthora *spp. (*P. infestans* and *P. palmivora*) [58]. Recently, Jeon and colleagues [55] carried out large-scale insertional mutagenesis of the *M. oryzae *strain KJ201 via ATMT to identify pathogenicity genes. They obtained 21,070 hygromycin-resistant mutants, which were tagged with T-DNA. Over 80% of the mutants were estimated to have a single copy of the T-DNA integrated into the genome. These mutants were then screened to detect disruption of seven phenotypic characters, such as fungal growth, pigmentation, conidiation, conidial morphology, conidial germination, appressorium formation, and pathogenicity. ATMT has been shown to have several advantages over conventional transformation methods like CaCl_2_/PEG-mediated transformation, lithium acetate-mediated transformation, particle bombardment, and electroporation. The principal advantage of ATMT over conventional transformation techniques is its versatility in choosing which starting material to transform [44]. Intact cells, such as conidia and mycelia, can be used as starting material, thereby eliminating the need to generate protoplasts. ATMT results in higher transformation efficiencies when compared with the above-mentioned transformation methods. T-DNA is an efficient substrate for homologous recombination, leading to relatively high gene knock-out frequencies. Another key advantage is that it generates a high percentage of transformants with a single copy insert of DNA, which facilitates the isolation of tagged genes [45]. These merits make ATMT a valuable tool to perform global or systematic mutational analyses in phytopathogens, either by targeted or insertional mutagenesis. ATMT is less suitable for generating strains for high protein production, due to largely single-copy T-DNA integration, whereas multiple gene copies are usually required for higher expression levels [59, 60].

The second insertional mutagenesis technique, REMI, can be used to generate random and targeted insertional mutations in phytopathogens. In REMI mutagenesis, linearized plasmid DNA is integrated into fungal protoplast in the presence of a restriction enzyme used to linearize the vector. The restriction enzyme targets the nucleus and induces double stranded breaks in the genome. As a result, plasmid integration takes place at the corresponding restriction sites in the genome, by recombining the ends of these breakages with the linearized plasmid [61]. The REMI technique was originally developed for *S. cerevisiae* [62]. Although REMI is an efficient tool for tagging and cloning pathogenicity genes from phytotopathogens, a substantial portion (20 to 100%) of generated mutants appears to be untagged by the transforming DNA. In spite of this, REMI has remained a powerful genetic tool for the past 15 years and has been used to mutagenize and tag genes in several phytopathogen species, including *Cochliobolus heterostrophus* [63], *M. oryzae* [64–66],* U. maydis *[67], *Colletotrichum *spp. (*C. lindemuthianum* and* C. graminicola*) [68], and *Pyrenophora teres* [69].

The biggest merit of this technique can be a significantly higher percentage of single-copy integration. However, REMI has some limitations. It requires fungal protoplast preparation, which is time consuming and laborious. Yield and viability of protoplasts are dependent on enzyme batches used to digest fungal cell walls and their ability to digest cell walls from different phytopathogens [70, 71]. REMI can also generate a significant number of different integration events, including single insertion with deletion of flanking restriction sites, nonhomologous integration in the absence of an appropriate restriction site, tandem insertion, and large genome deletions or inversions [72–77].

### 2.4. Tilling

**Figure 4:**
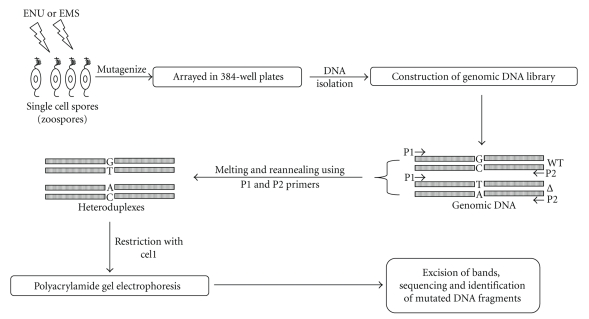
An illustrative TILLING strategy for phytopathogens. TILLING process encompasses the development and screening of a chemically mutagenized population from single cell uninucleated spores like zoospores to isolate individuals carrying induced mutations within the target gene.

The genome sequence drafts of the five oomycetes *Phytophthora sojae*, *P. ramorum*, *P. infestans*, and *P. capsici*, and *Hyaloperonospora parasitica* have been completed [78]. The next major undertaking is now to transcribe this genome information into biological function. McCallum and colleagues [79] introduced a new reverse genetic strategy for plants known as Targeting Induced Local Lesions In Genomes (TILLING) that combines the efficiency of ethyl methanesulfonate-(EMS-) induced mutagenesis (chemical mutagenesis) with the ability of denaturing high-performance liquid chromatography (DHPLC) to detect base pair changes (G/C to A/T transition) by heteroduplex analysis. TILLING has emerged as an influential tool for functional genomics of phytopathogens. Lamour and colleagues [80] employed TILLING to isolate gene-specific mutants in *Phytophthora* spp. They constructed a library of 2400 ethylnitrosourea (ENU) mutants of *P. sojae *and screened for induced point mutations in the genes encoding a necrosis-inducing protein (*PsojNIP*) and a *Phytophthora*-specific phospholipase D (*PsPXTMPLD*). Homozygous mutants carrying a potentially deleterious missense mutation in *PsojNIP *and a premature stop codon in *PsPXTM-PLD *were identified. No phenotypical changes were observed in *PsojNIP *mutants; however *PsPXTM-PLD *mutants showed reduced mycelial growth. Figure 4 shows an illustrative TILLING strategy. Single cell uninucleated spores like zoospores of *Phytophthora* spp. are ideal for TILLING mutagenesis. These spores are mutated by ENU or EMS and then arrayed into 384-well plates. A genomic DNA library is constructed using DNA extracted from individual mutant colonies followed by PCR amplification using sequence specific forward (P1) and reverse (P2) primers. Purified PCR products are then heated and cooled down to form heteroduplexes between wild type (WT) and mutant (Δ) DNA strands. The heteroduplexes are restricted with the single strand specific endonuclease Cel1, which cuts 3′ ends of single base mismatches producing novel DNA fragments. These fragments are resolved on a gel by polyacrylamide gel electrophoresis (PAGE). Fragment bands are then excised from the gel, purified and sequenced to identify mutant colonies carrying an induced point mutation. Confirmed mutants are characterized to determine the impact of mutation on the phenotype.

The main disadvantage of insertional mutagenesis is a relatively low mutation rate, which makes it difficult to tag a specific gene [81]. Unlike insertional mutagenesis, chemical mutagenesis like EMS treatment brings about a high mutation frequency without apparent preferences for specific genomic regions. This method can also generate many alleles, which facilitates the recovery of null phenotypes [82]. Although the development of novel mutagenesis techniques may eventually make TILLING obsolete, at present, it remains the technique of choice for medium- to high-throughput reverse genetics analyses in many phytopathogens [83].

## 3. Concluding Remarks

With the recent expansion of phytopathogen genome sequence data banks, locus-to-phenotype or gene-to-phenotype reverse genetic tools, such as knock-out, RNAi, ATMT, REMI, and TILLING, have become increasingly attractive methods to elucidate the molecular basis of host-pathogen interactions (compatible or incompatible), phytopathogen development, and virulence and pathogenicity. These reverse genetics tools can efficiently decode genome information into biological information. In the post-genomic era, gene targeting (knock-out) by homologous recombination has become the most influential reverse genetics tool to identify gene function. However, knock-out remains elusive for those phytopathogens that show low homologous recombination. Furthermore, the multinucleate nature of filamentous phytopathogens represents another challenge for knock-out as well as insertional mutageneses, which rely on the isolation of homokaryotic transformants derived from a single transformation to study loss of function/null mutants. RNAi, which disrupts gene expression by targeting the mRNA rather than the gene, may offer a solution to both problems [41]. However, the application of RNAi in filamentous phytopathogens is still in the developmental stage and does not work efficiently in every phytopathogen. The next challenges in RNAi will be the assessment of the extent of off-target effects in phytopathogens and the development of an inducible RNAi system coupled with a strictly controlled promoter and a convenient inducer that are applicable to a wide range of filamentous phytopathogens [42]. Gene tagging using insertional mutagenesis tools provides high transformation frequency and random insertion as a single copy insert. In this regard, ATMT has a clear advantage over REMI as it shows relatively higher transformation frequency and does not require protoplast preparation. TILLING certainly adds to the arsenal of reverse genetics tools, but it may become obsolete once new mutagenesis techniques have been developed. All the reverse genetics tools described above have their own merits and demerits, and any one of them may be more effective for a particular phytopathogen while less suitable for others. Therefore, a careful planning is required to harness the advantage of reverse genetics tools prior to conduct functional analyses.
